# The genome sequence of an ichneumonid wasp, *Perithous albicinctus* (Gravenhorst, 1829) (Hymenoptera: Ichneumonidae)

**DOI:** 10.12688/wellcomeopenres.24928.1

**Published:** 2025-10-03

**Authors:** Liam M. Crowley, James McCulloch, Augustijn De Ketelaere, Gavin R. Broad, Natural History Museum Genome Acquisition Lab, Darwin Tree of Life Barcoding Collective, Wellcome Sanger Institute Tree of Life Management, Samples and Laboratory team, Wellcome Sanger Institute Scientific Operations: Sequencing Operations, Wellcome Sanger Institute Tree of Life Core Informatics team, Tree of Life Core Informatics collective, Darwin Tree of Life Consortium

**Affiliations:** 1 University of Oxford, Oxford, England, UK; 2 Wellcome Sanger Institute, Hinxton, England, UK; 3 Independent researcher, Beernem, Flanders, Belgium; 4 Natural History Museum, London, England, UK

**Keywords:** Perithous albicinctus; ichneumonid wasp; genome sequence; chromosomal; Hymenoptera

## Abstract

We present a genome assembly from an individual female *Perithous albicinctus* (ichneumonid wasp; Arthropoda; Insecta; Hymenoptera; Ichneumonidae). The genome sequence has a total length of 383.46 megabases. Most of the assembly (97.06%) is scaffolded into 17 chromosomal pseudomolecules. The mitochondrial genome has also been assembled, with a length of 35.92 kilobases. This assembly was generated as part of the Darwin Tree of Life project, which produces reference genomes for eukaryotic species found in Britain and Ireland.

## Species taxonomy

Eukaryota; Opisthokonta; Metazoa; Eumetazoa; Bilateria; Protostomia; Ecdysozoa; Panarthropoda; Arthropoda; Mandibulata; Pancrustacea; Hexapoda; Insecta; Dicondylia; Pterygota; Neoptera; Endopterygota; Hymenoptera; Apocrita; Ichneumonoidea; Ichneumonidae; Pimplinae; Perithoini; *Perithous*; *Perithous albicinctus* (Gravenhorst, 1829) (NCBI:txid2998544)

## Background

*Perithous albicinctus* is a relatively large (fore wing length ~12 mm) ichneumonid wasp with a long ovipositor, mostly black but with some pale yellowish markings on the head and thorax. The male has an entirely yellow face whereas the female has just the eye orbits yellow. The genus *Perithous* can be identified and separated from various other genera of Pimplinae with long ovipositors using the key in Fitton *et al*. (1988). British *Perithous* can be identified using Shaw (2006) or, globally, using Gupta (1982).

*Perithous* species are parasitoids of solitary stem-nesting apoid wasps, attacking the larvae in their nests. They are idiobiont ectoparasitoids, i.e., the host is permanently paralysed and feeding is external, therefore physiologically relatively unspecialised, so other parasitoid larvae which attacked the aculeate host can be eaten by the *Perithous* larva ( Danks, 1971). The known hosts of *P. albicinctus* are relatively large Crabronidae of the genus *Ectemnius* ( Aubert, 1969; e.g., Faester, 1944). *Perithous albicinctus* is found across the Palaearctic, as far East as Japan ( Watanabe & Yamauchi, 2014). Because of its large size and distinctive appearance, it is unlikely that *P. albicinctus* was overlooked in Britain, and it had not been found when the British handbook for the identification of British pimplines ( Fitton *et al*., 1988) was published. We can assume that this was a recent colonist when first recorded from England by Brock & Shaw (1997). Since the 1990s, *P. albicinctus* has spread across much of southern England, reaching Yorkshire by 2004 ( Mayhew *et al*., 2009). There is probably considerable change in parasitoid faunas, but it is rare to have the opportunity to track range expansion.

The small pimpline tribe Delomeristini, comprising the genera *Atractogaster*, *Delomerista, Perithous* and *Pseudorhyssa*, are almost all parasitoids of other Hymenoptera ( Broad *et al*., 2018; Gauld *et al*., 2002), and this biological trait has tended to be more reliable for grouping close relatives than morphology, with these genera bouncing around between different tribes until Klopfstein *et al*. (2019) employed a large molecular dataset to resolve many of the higher level relationships in Pimplinae and relatives. Interestingly, the results of Klopfstein *et al*. (2019) are very close to the relationships proposed by Townes (1969) ‘intuitively’, on the basis of his understanding of ichneumonid biology and morphology. The availability of an increasing number of genomes of Pimplinae gives new opportunities to test hypotheses around the evolution of the diverse host range of the Pimplinae Wahl & Gauld (1998), a group which seemed to diversify rapidly ( Klopfstein *et al*., 2019).

**Figure 1.**
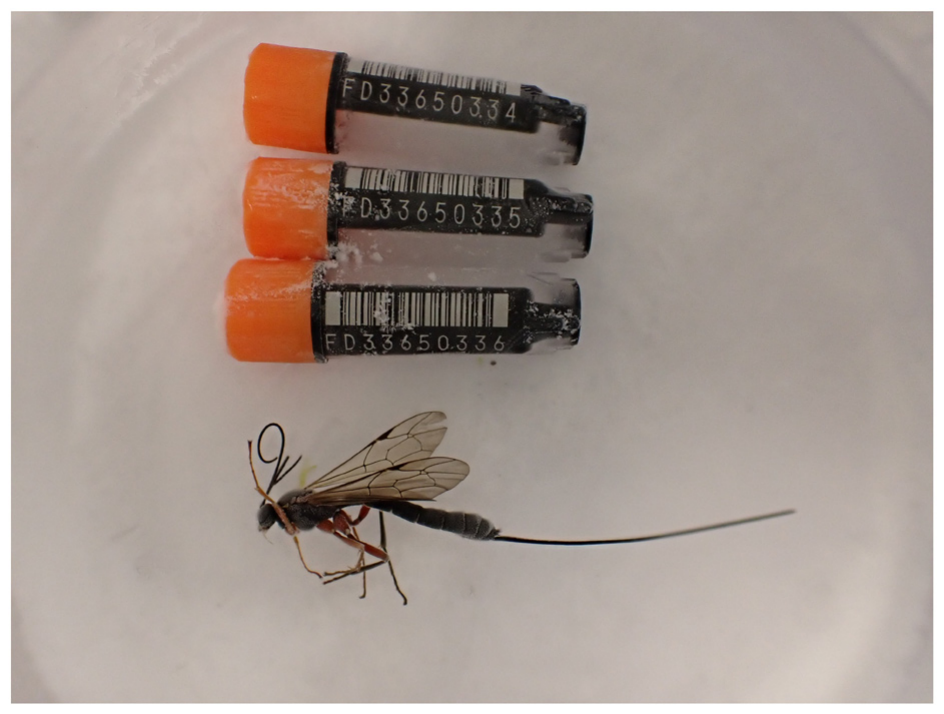
Photograph of the *Perithous albicinctus* (iyPerAlbc1) specimen used for genome sequencing.

We present a chromosome-level genome sequence for *Perithous albicinctus*, the first genome for the genus *Perithous* (data obtained via NCBI datasets, O’Leary *et al*., 2024). The assembly was produced using the Tree of Life pipeline from a specimen collected in Wytham Woods, Oxfordshire, United Kingdom ( Figure 1).

## Methods

### Sample acquisition and DNA barcoding

The specimen used for genome sequencing was an adult female *Perithous albicinctus* (specimen ID Ox002617, ToLID iyPerAlbc1; Figure 1), collected from Wytham Woods, Oxfordshire, United Kingdom (latitude 51.772, longitude –1.338) on 2022-08-02. The specimen was collected by James McCulloch and Liam Crowley and formally identified by Augustijn De Ketelaere. For the Darwin Tree of Life sampling and metadata approach, refer to Lawniczak *et al*. (2022).

The initial identification was verified by an additional DNA barcoding process according to the framework developed by Twyford *et al.* (2024). A small sample was dissected from the specimen and stored in ethanol, while the remaining parts were shipped on dry ice to the Wellcome Sanger Institute (WSI) (see the protocol). The tissue was lysed, the COI marker region was amplified by PCR, and amplicons were sequenced and compared to the BOLD database, confirming the species identification ( Crowley *et al*., 2023). Following whole genome sequence generation, the relevant DNA barcode region was also used alongside the initial barcoding data for sample tracking at the WSI ( Twyford *et al*., 2024). The standard operating procedures for Darwin Tree of Life barcoding are available on protocols.io.

### Nucleic acid extraction

Protocols for high molecular weight (HMW) DNA extraction developed at the Wellcome Sanger Institute (WSI) Tree of Life Core Laboratory are available on protocols.io ( Howard *et al*., 2025). The iyPerAlbc1 sample was weighed and triaged to determine the appropriate extraction protocol. Tissue from the thorax was homogenised by powermashing using a PowerMasher II tissue disruptor.

HMW DNA was extracted in the WSI Scientific Operations core using the Automated MagAttract v2 protocol. DNA was sheared into an average fragment size of 12–20 kb following the Megaruptor®3 for LI PacBio protocol. Sheared DNA was purified by manual SPRI (solid-phase reversible immobilisation). The concentration of the sheared and purified DNA was assessed using a Nanodrop spectrophotometer and Qubit Fluorometer using the Qubit dsDNA High Sensitivity Assay kit. Fragment size distribution was evaluated by running the sample on the FemtoPulse system. For this sample, the final post-shearing DNA had a Qubit concentration of 18 ng/μL and a yield of 810.00 ng, with a fragment size of 11.7 kb. The 260/280 spectrophotometric ratio was 2.24, and the 260/230 ratio was 1.57.

### PacBio HiFi library preparation and sequencing

Library preparation and sequencing were performed at the WSI Scientific Operations core. Libraries were prepared using the SMRTbell Prep Kit 3.0 (Pacific Biosciences, California, USA), following the manufacturer’s instructions. The kit includes reagents for end repair/A-tailing, adapter ligation, post-ligation SMRTbell bead clean-up, and nuclease treatment. Size selection and clean-up were performed using diluted AMPure PB beads (Pacific Biosciences). DNA concentration was quantified using a Qubit Fluorometer v4.0 (ThermoFisher Scientific) and the Qubit 1X dsDNA HS assay kit. Final library fragment size was assessed with the Agilent Femto Pulse Automated Pulsed Field CE Instrument (Agilent Technologies) using the gDNA 55 kb BAC analysis kit.

The sample was sequenced using the Sequel IIe system (Pacific Biosciences, California, USA). The concentration of the library loaded onto the Sequel IIe was in the range 40–135 pM. The SMRT link software, a PacBio web-based end-to-end workflow manager, was used to set-up and monitor the run, and to perform primary and secondary analysis of the data upon completion.

### Hi-C

***Sample preparation and crosslinking***

The Hi-C sample was prepared from 20–50 mg of frozen head tissue from the iyPerAlbc1 sample using the Arima-HiC v2 kit (Arima Genomics). Following the manufacturer’s instructions, tissue was fixed and DNA crosslinked using TC buffer to a final formaldehyde concentration of 2%. The tissue was homogenised using the Diagnocine Power Masher-II. Crosslinked DNA was digested with a restriction enzyme master mix, biotinylated, and ligated. Clean-up was performed with SPRISelect beads before library preparation. DNA concentration was measured with the Qubit Fluorometer (Thermo Fisher Scientific) and Qubit HS Assay Kit. The biotinylation percentage was estimated using the Arima-HiC v2 QC beads.

***Hi-C library preparation and sequencing***

Biotinylated DNA constructs were fragmented using a Covaris E220 sonicator and size selected to 400–600 bp using SPRISelect beads. DNA was enriched with Arima-HiC v2 kit Enrichment beads. End repair, A-tailing, and adapter ligation were carried out with the NEBNext Ultra II DNA Library Prep Kit (New England Biolabs), following a modified protocol where library preparation occurs while DNA remains bound to the Enrichment beads. Library amplification was performed using KAPA HiFi HotStart mix and a custom Unique Dual Index (UDI) barcode set (Integrated DNA Technologies). Depending on sample concentration and biotinylation percentage determined at the crosslinking stage, libraries were amplified with 10–16 PCR cycles. Post-PCR clean-up was performed with SPRISelect beads. Libraries were quantified using the AccuClear Ultra High Sensitivity dsDNA Standards Assay Kit (Biotium) and a FLUOstar Omega plate reader (BMG Labtech).

Prior to sequencing, libraries were normalised to 10 ng/μL. Normalised libraries were quantified again and equimolar and/or weighted 2.8 nM pools. Pool concentrations were checked using the Agilent 4200 TapeStation (Agilent) with High Sensitivity D500 reagents before sequencing. Sequencing was performed using paired-end 150 bp reads on the Illumina NovaSeq 6000.

### Genome assembly

Prior to assembly of the PacBio HiFi reads, a database of *k*-mer counts ( *k* = 31) was generated from the filtered reads using FastK. GenomeScope2 ( Ranallo-Benavidez *et al*., 2020) was used to analyse the *k*-mer frequency distributions, providing estimates of genome size, heterozygosity, and repeat content.

The HiFi reads were assembled using Hifiasm ( Cheng *et al*., 2021) with the --primary option. Haplotypic duplications were identified and removed using purge_dups ( Guan *et al*., 2020). The Hi-C reads ( Rao *et al*., 2014) were mapped to the primary contigs using bwa-mem2 ( Vasimuddin *et al*., 2019), and the contigs were scaffolded in YaHS ( Zhou *et al*., 2023) with the --break option for handling potential misassemblies. The scaffolded assemblies were evaluated using Gfastats ( Formenti *et al*., 2022), BUSCO ( Manni *et al*., 2021) and MERQURY.FK ( Rhie *et al*., 2020).

The mitochondrial genome was assembled using MitoHiFi ( Uliano-Silva *et al*., 2023), which runs MitoFinder ( Allio *et al*., 2020) and uses these annotations to select the final mitochondrial contig and to ensure the general quality of the sequence.

### Assembly curation

The assembly was decontaminated using the Assembly Screen for Cobionts and Contaminants ( ASCC) pipeline. TreeVal was used to generate the flat files and maps for use in curation. Manual curation was conducted primarily in PretextView and HiGlass ( Kerpedjiev *et al*., 2018). Scaffolds were visually inspected and corrected as described by Howe *et al*. (2021). Manual corrections included 21 breaks, 136 joins, and removal of 5 haplotypic duplications. The curation process is documented at https://gitlab.com/wtsi-grit/rapid-curation. PretextSnapshot was used to generate a Hi-C contact map of the final assembly.

### Assembly quality assessment

The Merqury.FK tool ( Rhie *et al*., 2020) was run in a Singularity container ( Kurtzer *et al*., 2017) to evaluate *k*-mer completeness and assembly quality for the primary and alternate haplotypes using the *k*-mer databases ( *k* = 31) computed prior to genome assembly. The analysis outputs included assembly QV scores and completeness statistics.

The genome was analysed using the BlobToolKit pipeline, a Nextflow implementation of the earlier Snakemake version ( Challis *et al*., 2020). The pipeline aligns PacBio reads using minimap2 ( Li, 2018) and SAMtools ( Danecek *et al*., 2021) to generate coverage tracks. It runs BUSCO ( Manni *et al*., 2021) using lineages identified from the NCBI Taxonomy ( Schoch *et al*., 2020). For the three domain-level lineages, BUSCO genes are aligned to the UniProt Reference Proteomes database ( Bateman *et al*., 2023) using DIAMOND blastp ( Buchfink *et al*., 2021). The genome is divided into chunks based on the density of BUSCO genes from the closest taxonomic lineage, and each chunk is aligned to the UniProt Reference Proteomes database with DIAMOND blastx. Sequences without hits are chunked using seqtk and aligned to the NT database with blastn ( Altschul *et al*., 1990). The BlobToolKit suite consolidates all outputs into a blobdir for visualisation. The BlobToolKit pipeline was developed using nf-core tooling ( Ewels *et al*., 2020) and MultiQC ( Ewels *et al*., 2016), with containerisation through Docker ( Merkel, 2014) and Singularity ( Kurtzer *et al*., 2017).

## Genome sequence report

### Sequence data

**Figure 2.**
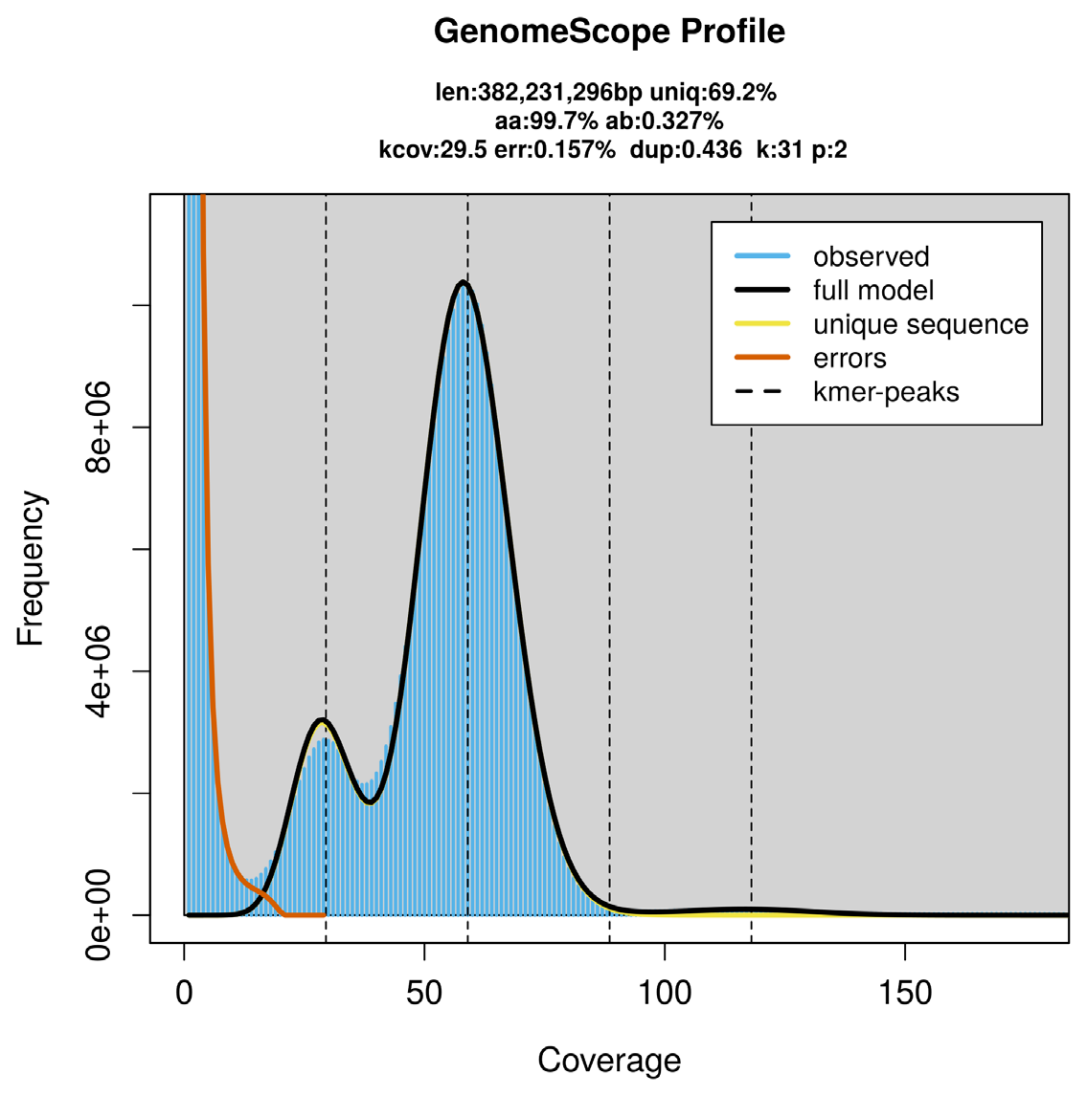
Frequency distribution of *k*-mers generated using GenomeScope2. The plot shows observed and modelled *k*-mer spectra, providing estimates of genome size, heterozygosity, and repeat content based on unassembled sequencing reads.

PacBio sequencing of the *Perithous albicinctus* specimen generated 23.75 Gb (gigabases) from 2.45 million reads, which were used to assemble the genome. GenomeScope2.0 analysis estimated the haploid genome size at 382.23 Mb, with a heterozygosity of 0.33% and repeat content of 30.89% ( Figure 2). These estimates guided expectations for the assembly. Based on the estimated genome size, the sequencing data provided approximately 59× coverage. Hi-C sequencing produced 109.26 Gb from 723.60 million reads, which were used to scaffold the assembly. Table 1 summarises the specimen and sequencing details.

**Table 1.** Specimen and sequencing data for BioProject PRJEB64967.

Platform	PacBio HiFi	Hi-C
**ToLID**	iyPerAlbc1	iyPerAlbc1
**Specimen ID**	Ox002617	Ox002617
**BioSample (source individual)**	SAMEA112232798	SAMEA112232798
**BioSample (tissue)**	SAMEA112233302	SAMEA112233301
**Tissue**	thorax	head
**Instrument**	Sequel IIe	Illumina NovaSeq 6000
**Run accessions**	ERR11843436	ERR11837526
**Read count total**	2.45 million	723.60 million
**Base count total**	23.75 Gb	109.26 Gb

### Assembly statistics

The primary haplotype was assembled, and contigs corresponding to an alternate haplotype were also deposited in INSDC databases. The final assembly has a total length of 383.46 Mb in 49 scaffolds, with 395 gaps, and a scaffold N50 of 24.66 Mb ( Table 2).

**Table 2.** Genome assembly statistics.

**Assembly name**	iyPerAlbc1.1
**Assembly accession**	GCA_964016905.1
**Alternate haplotype accession**	GCA_964016955.1
**Assembly level**	chromosome
**Span (Mb)**	383.46
**Number of chromosomes**	17
**Number of contigs**	444
**Contig N50**	1.86 Mb
**Number of scaffolds**	49
**Scaffold N50**	24.66 Mb
**Organelles**	Mitochondrion: 35.92 kb

**Figure 3.**
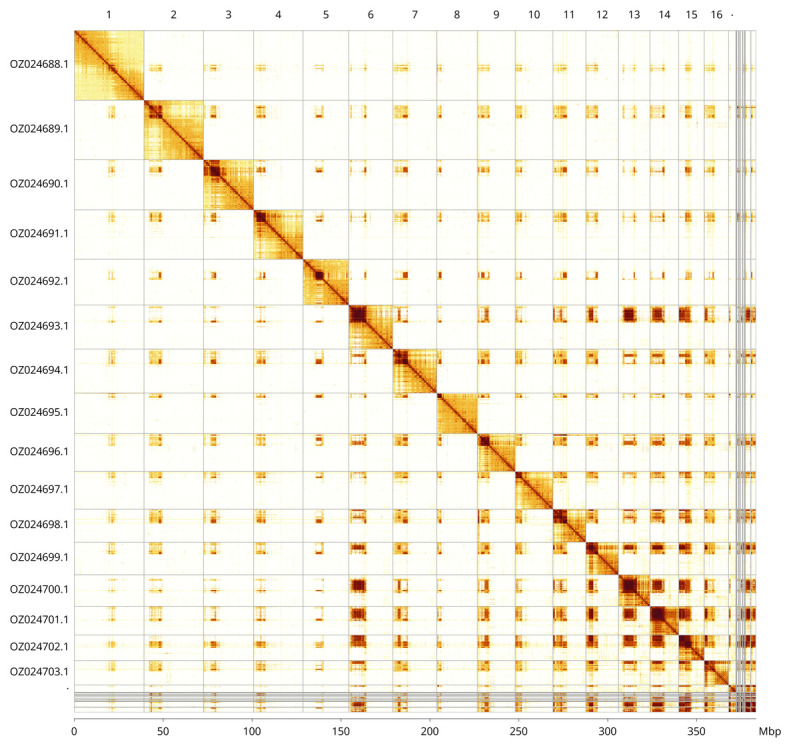
Hi-C contact map of the *Perithous albicinctus* genome assembly. Assembled chromosomes are shown in order of size and labelled along the axes. The plot was generated using PretextSnapshot.

Most of the assembly sequence (97.06%) was assigned to 17 chromosomal-level scaffolds. These chromosome-level scaffolds, confirmed by Hi-C data, are named according to size ( Figure 3; Table 3). Scaffolds in the following regions have uncertain order and orientation: Chromosome 4 between approximately 1.8–4.3 Mb, Chromosome 6 between approximately 2.7–8.8 Mb, Chromosome 9 between approximately 2.3–6.8 Mb, and Chromosome 15 between approximately 0–7 Mb.

**Table 3.** Chromosomal pseudomolecules in the primary genome assembly of *Perithous albicinctus* iyPerAlbc1.

INSDC accession	Molecule	Length (Mb)	GC%
OZ024688.1	1	39.28	36
OZ024689.1	2	33.46	37
OZ024690.1	3	28.24	37
OZ024691.1	4	27.77	37
OZ024692.1	5	25.71	37
OZ024693.1	6	24.83	37.50
OZ024694.1	7	24.66	38
OZ024695.1	8	22.84	36.50
OZ024696.1	9	21.30	37.50
OZ024697.1	10	21.14	37
OZ024698.1	11	18.57	38
OZ024699.1	12	18.30	38
OZ024700.1	13	17.74	37.50
OZ024701.1	14	16.17	39
OZ024702.1	15	14.30	39
OZ024703.1	16	13.80	37.50
OZ024704.1	17	4.09	37.50

The mitochondrial genome was also assembled. This sequence is included as a contig in the multifasta file of the genome submission and as a standalone record.

**Figure 4.**
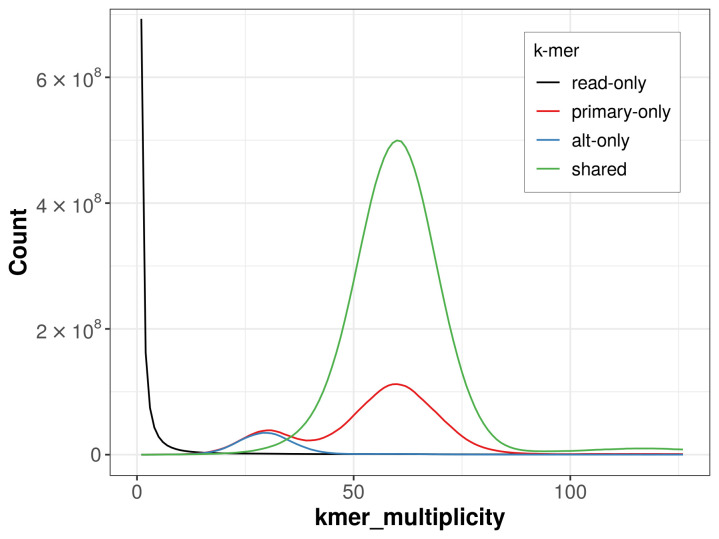
Evaluation of *k*-mer completeness using MerquryFK. This plot illustrates the recovery of *k*‐mers from the original read data in the final assemblies. The horizontal axis represents *k*‐mer multiplicity, and the vertical axis shows the number of *k*‐mers. The black curve represents *k*‐mers that appear in the reads but are not assembled. The green curve corresponds to *k*‐mers shared by both haplotypes, and the red and blue curves show *k*‐mers found only in one of the haplotypes.

The combined primary and alternate assemblies achieve an estimated QV of 60.9. The *k*-mer completeness is 92.52% for the primary assembly, 77.08% for the alternate haplotype, and 98.97% for the combined assemblies ( Figure 4).

**Figure 5.**
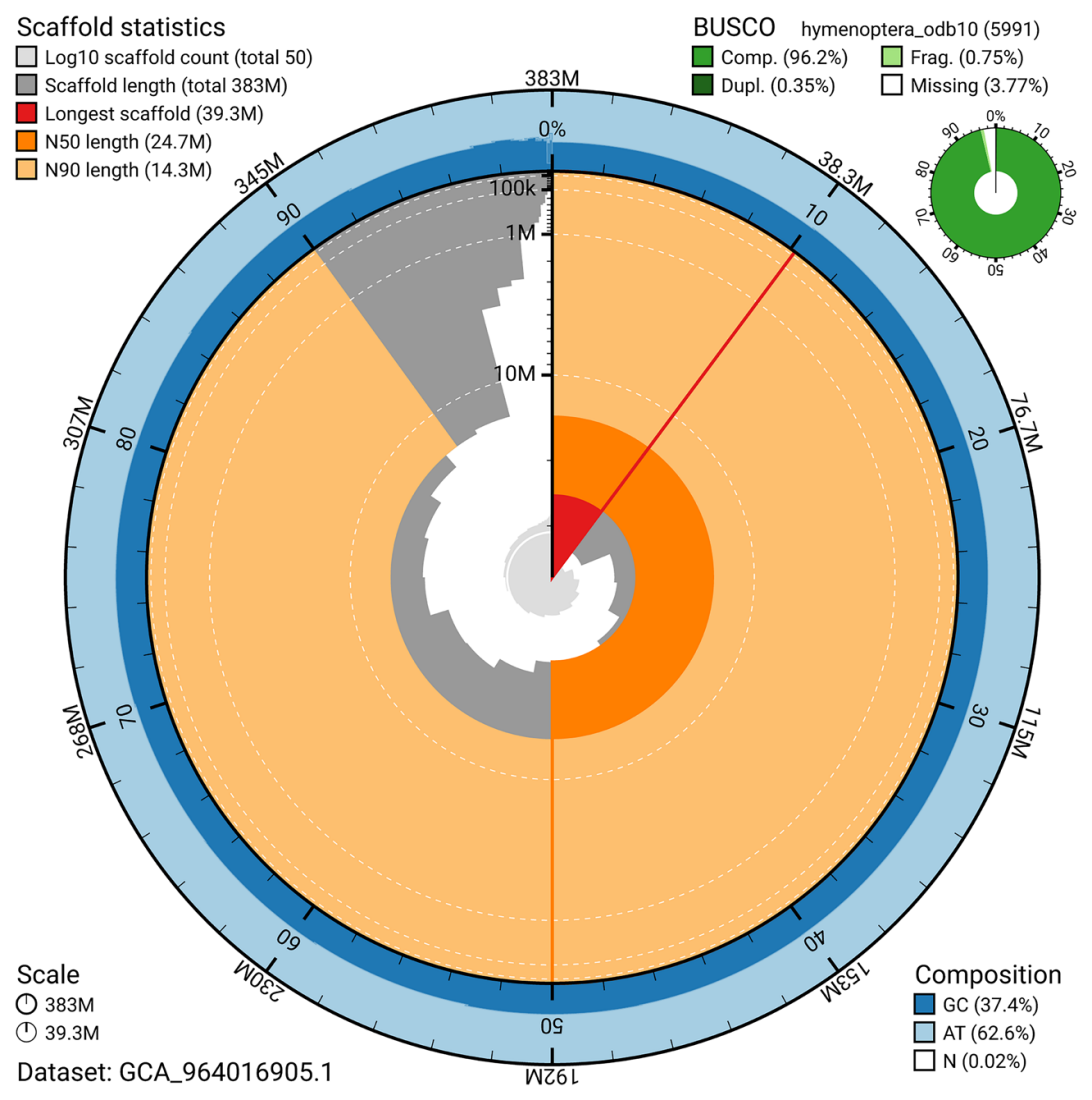
Assembly metrics for iyPerAlbc1.1. The BlobToolKit snail plot provides an overview of assembly metrics and BUSCO gene completeness. The circumference represents the length of the whole genome sequence, and the main plot is divided into 1 000 bins around the circumference. The outermost blue tracks display the distribution of GC, AT, and N percentages across the bins. Scaffolds are arranged clockwise from longest to shortest and are depicted in dark grey. The longest scaffold is indicated by the red arc, and the deeper orange and pale orange arcs represent the N50 and N90 lengths. A light grey spiral at the centre shows the cumulative scaffold count on a logarithmic scale. A summary of complete, fragmented, duplicated, and missing BUSCO genes in the hymenoptera_odb10 set is presented at the top right. An interactive version of this figure can be accessed on the BlobToolKit viewer.

**Figure 6.**
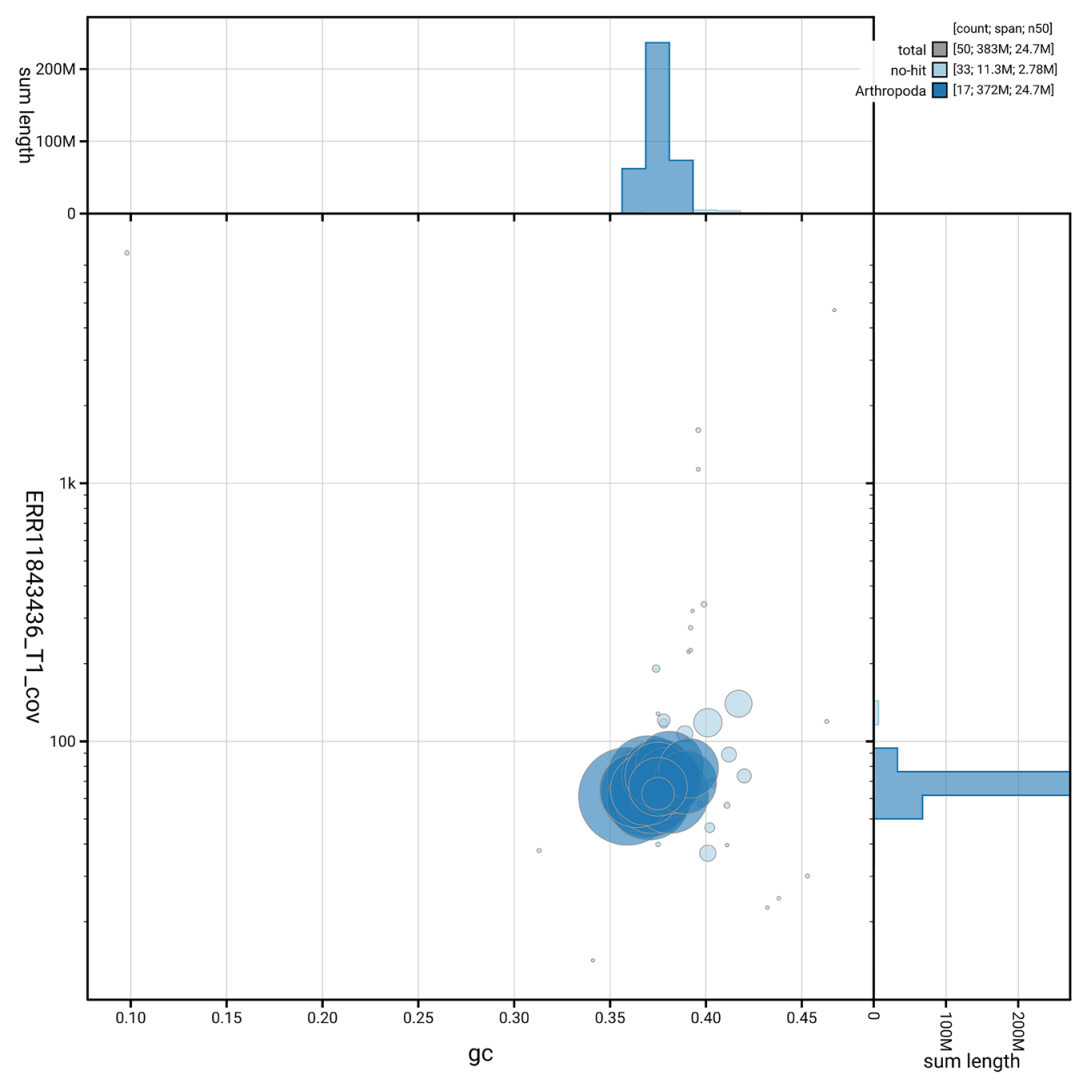
BlobToolKit GC-coverage plot for iyPerAlbc1.1. Blob plot showing sequence coverage (vertical axis) and GC content (horizontal axis). The circles represent scaffolds, with the size proportional to scaffold length and the colour representing phylum membership. The histograms along the axes display the total length of sequences distributed across different levels of coverage and GC content. An interactive version of this figure is available on the BlobToolKit viewer.

BUSCO v.5.5.0 analysis using the hymenoptera_odb10 reference set ( *n* = 5 991) identified 96.2% of the expected gene set (single = 95.9%, duplicated = 0.4%). The snail plot in Figure 5 summarises the scaffold length distribution and other assembly statistics for the primary assembly. The blob plot in Figure 6 shows the distribution of scaffolds by GC proportion and coverage.

Table 4 lists the assembly metric benchmarks adapted from Rhie *et al.* (2021) the Earth BioGenome Project Report on Assembly Standards September 2024. The EBP metric, calculated for the primary assembly, is **6.C.Q60**, meeting the recommended reference standard.

**Table 4.** Earth Biogenome Project summary metrics for the *Perithous albicinctus* assembly.

Measure	Value	Benchmark
EBP summary (primary)	6.C.Q60	6.C.Q40
Contig N50 length	1.86 Mb	≥ 1 Mb
Scaffold N50 length	24.66 Mb	= chromosome N50
Consensus quality (QV)	Primary: 60.9; alternate: 60.9; combined: 60.9	≥ 40
*k*-mer completeness	Primary: 92.52%; alternate: 77.08%; combined: 98.97%	≥ 95%
BUSCO	C:96.2% [S:95.9%; D:0.4%]; F:0.8%; M:3.0%; n:5 991	S > 90%; D < 5%
Percentage of assembly assigned to chromosomes	97.06%	≥ 90%

### Wellcome Sanger Institute – Legal and Governance

The materials that have contributed to this genome note have been supplied by a Darwin Tree of Life Partner. The submission of materials by a Darwin Tree of Life Partner is subject to the **‘Darwin Tree of Life Project Sampling Code of Practice’**, which can be found in full on the Darwin Tree of Life website. By agreeing with and signing up to the Sampling Code of Practice, the Darwin Tree of Life Partner agrees they will meet the legal and ethical requirements and standards set out within this document in respect of all samples acquired for, and supplied to, the Darwin Tree of Life Project. Further, the Wellcome Sanger Institute employs a process whereby due diligence is carried out proportionate to the nature of the materials themselves, and the circumstances under which they have been/are to be collected and provided for use. The purpose of this is to address and mitigate any potential legal and/or ethical implications of receipt and use of the materials as part of the research project, and to ensure that in doing so we align with best practice wherever possible. The overarching areas of consideration are:

- Ethical review of provenance and sourcing of the material
- Legality of collection, transfer and use (national and international)

Each transfer of samples is further undertaken according to a Research Collaboration Agreement or Material Transfer Agreement entered into by the Darwin Tree of Life Partner, Genome Research Limited (operating as the Wellcome Sanger Institute), and in some circumstances, other Darwin Tree of Life collaborators.

## Data Availability

European Nucleotide Archive: Perithous albicinctus. Accession number PRJEB64967. The genome sequence is released openly for reuse. The *Perithous albicinctus* genome sequencing initiative is part of the Darwin Tree of Life Project (PRJEB40665) and the Sanger Institute Tree of Life Programme (PRJEB43745). All raw sequence data and the assembly have been deposited in INSDC databases. The genome will be annotated using available RNA-Seq data and presented through the Ensembl pipeline at the European Bioinformatics Institute. Raw data and assembly accession identifiers are reported in Table 1 and Table 2. Production code used in genome assembly at the WSI Tree of Life is available at https://github.com/sanger-tol. Table 5 lists software versions used in this study.

## Author information

Contributors are listed at the following links:

- Members of the University of Oxford and Wytham Woods Genome Acquisition Lab
- Members of the Darwin Tree of Life Barcoding collective
- Members of the Wellcome Sanger Institute Tree of Life Management, Samples and Laboratory team
- Members of Wellcome Sanger Institute Scientific Operations – Sequencing Operations
- Members of the Wellcome Sanger Institute Tree of Life Core Informatics team
- Members of the Tree of Life Core Informatics collective
- Members of the Darwin Tree of Life Consortium
