# Supplementary material for: The genome sequence of an ichneumonid wasp, Perithous albicinctus (Gravenhorst, 1829) (Hymenoptera: Ichneumonidae)
Source: Wellcome Open Res. 2025 Oct 3;10:540. [Version 1] doi: 10.12688/wellcomeopenres.24928.1 (PMC12648025; doi:10.12688/wellcomeopenres.24928.1)
Supplement: Peer review history

# Reviewer report: Reviewer response for version 1

**DOI:** 10.21956/wellcomeopenres.27458.r140151

**Published:** 2025-12-29

**Authors:** Sara E Miller

**Affiliations:** 1 Biology, University of Missouri St. Louis (Ringgold ID: 2628), St. Louis, Missouri, USA

The authors present a new high quality nuclear and mitochondrial genome assembly for the ichneumonid wasp, *Perithous albicinctus* ****(Hymenoptera: Ichneumonidae) in the tribe Delomeristini. Notably, this species appears to be a recent invasive species to England and since it was first detected, as subsequently spread across southern England. This is the first genome assembly for this species.

 The methods are well described, consistent with other genome assemblies from the Darwin Tree of Life project and exceed Earth BioGenome Project Assembly Standards benchmarks.  The final assembly is typical of Hymenoptera genome assembly. I recommend Indexing.

Are sufficient details of methods and materials provided to allow replication by others?

Yes

Is the rationale for creating the dataset(s) clearly described?

Yes

Are the datasets clearly presented in a useable and accessible format?

Yes

Are the protocols appropriate and is the work technically sound?

Yes

Reviewer Expertise:

Hymenoptera genetics and genomics, evolutionary ecology

I confirm that I have read this submission and believe that I have an appropriate level of expertise to confirm that it is of an acceptable scientific standard.

# Reviewer report: Reviewer response for version 1

**DOI:** 10.21956/wellcomeopenres.27458.r140147

**Published:** 2025-11-25

**Authors:** Jason Charamis

**Affiliations:** 1 Foundation for Research and Technology - Hellas, Irákleion, Greece

This manuscript describes the genome sequencing and assembly of the ichneumonid wasp *Perithous albicinctus . *The assembly is of good quality, while the                         manuscript provides a good description of the relevance of having an assembly of this species and the methodologies used to produce it. I have no comments or requests to make: I think the manuscript is ready for publication.

Are sufficient details of methods and materials provided to allow replication by others?

Yes

Is the rationale for creating the dataset(s) clearly described?

Yes

Are the datasets clearly presented in a useable and accessible format?

Yes

Are the protocols appropriate and is the work technically sound?

Yes

Reviewer Expertise:

Arthropod Comparative Genomics

I confirm that I have read this submission and believe that I have an appropriate level of expertise to confirm that it is of an acceptable scientific standard.

# Reviewer report: Reviewer response for version 1

**DOI:** 10.21956/wellcomeopenres.27458.r135256

**Published:** 2025-10-09

**Authors:** Hasin Ullah

**Affiliations:** 1 Northwest University, Xi'an, Shaanxi, China

**Comments:** The Methods and Materials are described in exceptional detail, covering all critical steps from specimen collection and DNA extraction to sequencing, assembly, and quality assessment. Each stage includes information on instruments, kits, software versions, parameters, and references to established protocols (e.g., Lawniczak et al., 2022; Twyford et al., 2024). The inclusion of accession numbers, precise laboratory procedures, and links to protocols.io further ensures reproducibility. Overall, the methodological transparency meets and even exceeds standard requirements for genome sequencing papers.

 The manuscript presents a technically robust genome assembly of *Perithous albicinctus*, generated under the Darwin Tree of Life framework. The sequencing depth, assembly metrics, and completeness all indicate a high-quality reference genome.

Are sufficient details of methods and materials provided to allow replication by others?

Yes

Is the rationale for creating the dataset(s) clearly described?

Yes

Are the datasets clearly presented in a useable and accessible format?

Yes

Are the protocols appropriate and is the work technically sound?

Yes

Reviewer Expertise:

Molecular phylogenetics

I confirm that I have read this submission and believe that I have an appropriate level of expertise to confirm that it is of an acceptable scientific standard.
